# Evaluating the clinical utility of iodine-123 scans in follow-up for differentiated thyroid cancer: a single-centre study

**DOI:** 10.1530/EO-25-0105

**Published:** 2026-04-08

**Authors:** Robert Smith-Baker, Richard Meades, Arunansu Kar

**Affiliations:** ^1^ The Royal Free NHS Foundation Trust, London, United Kingdom; ^2^ Department of Nuclear Medicine, The Royal Free NHS Foundation Trust, London, United Kingdom; ^3^ Department of Clinical Oncology, The Royal Free NHS Foundation Trust, London, United Kingdom

**Keywords:** thyroid, oncology, radiotherapy

## Abstract

**Objective:**

To evaluate the impact of iodine-123 (I-123) diagnostic scintigraphy on management outcomes in patients undergoing follow-up for differentiated thyroid carcinoma (DTC), and to assess whether multidisciplinary team (MDT) discussion influences subsequent clinical pathways.

**Methods:**

A retrospective analysis was conducted of patients with DTC who underwent I-123 scintigraphy at an NHS trust. Demographic data, scan findings, and subsequent clinical actions were collected. Scans were categorised as MDT endorsed or requested by individual clinicians. Outcomes were assessed by distinguishing initial diagnostic actions prompted by I-123 imaging from definitive management outcomes following completion of downstream investigations.

**Results:**

A total of 55 I-123 scans from 51 patients were included. New or abnormal findings were identified in 21 scans (38.2%). Initial diagnostic actions followed 23 scans (41.8%), most commonly further imaging. When management outcomes were reassessed after completion of downstream investigations, no definitive change in management occurred in 40 scans (72.7%), while 13 scans (23.6%) resulted in a definitive management change; in 2 scans (3.6%), outcomes were unclear. MDT-endorsed scans more frequently prompted initial diagnostic actions, although this difference was not statistically significant. Outcomes for MDT-requested scans and individually requested scans were near-identical (definitive change in management in 20% vs 20.7).

**Conclusions:**

I-123 scintigraphy can influence clinical decision-making during follow-up of patients with differentiated thyroid cancer, most commonly by prompting further diagnostic investigation. However, definitive changes in management occur in a minority of cases. These findings support a selective rather than routine role for I-123 imaging in follow-up.

## Introduction

Thyroid cancer ranks among the most prevalent endocrinological malignancies and is the fastest growing cancer worldwide (1). In the United Kingdom, thyroid cancer accounts for just over 1% of new cancer diagnoses and generally carries a favourable prognosis, with differentiated thyroid cancer (DTC) – papillary and follicular subtypes – achieving 10-year survival rates of around 85% (2, 3).

The treatment for DTC is well established. In the United Kingdom, tumours ≥4 cm, those with regional nodal involvement, or those with adverse pathological features are typically managed with total thyroidectomy and often followed by radioiodine-131 (I-131) ablation, in line with national guidance (4). This approach aligns with the best practice in the United States and Europe (5, 6). Ongoing follow-up relies mainly on serial thyroglobulin measurement and ultrasound, while diagnostic radioiodine imaging is reserved for selected cases (4).

I-131 has long been used for post-treatment ‘exit’ scans to detect residual thyroid tissue or metastatic disease (7). However, it is challenging to obtain artefact-free diagnostic image quality from its high-energy gamma photon. In contrast, iodine-123 (I-123) is a gamma emitter with photon energy better suited to gamma camera imaging, producing excellent image quality (8). Studies have demonstrated I-123 scans to be effective in imaging thyroid malignancy both before and after radioiodine therapy (7, 9, 10). Nevertheless, its cost, need for thyroid hormone withdrawal or recombinant human thyrotropin (rhTSH), and limited explicit guideline endorsement have restricted routine use.

Recent work, such as that by Campennì and colleagues, has helped identify situations where I-123 may be most useful, although much of the evidence addresses early post-therapy settings (11). Current UK national guidance offers no specific recommendations for I-123 in routine follow-up, and practice varies between centres and clinicians (4). This variability highlights the need to evaluate how I-123 scanning impacts downstream management decisions and whether multidisciplinary team (MDT) discussion enhances its clinical value. MDTs are integral in cancer care, improving diagnostic accuracy, treatment planning, and coordination between disciplines. Assessing how MDT endorsement influences I-123 imaging pathways after thyroidectomy may help refine investigation strategies and resource utilisation in DTC follow-up.

## Methods

### Study design and setting

This was a retrospective service evaluation conducted at a tertiary thyroid cancer and nuclear medicine centre. The study included all patients with histologically confirmed differentiated thyroid cancer who underwent I-123 diagnostic scintigraphy as part of routine clinical follow-up between January 2009 and December 2023.

### Ethics and governance

This project was undertaken as a service evaluation of established clinical practice. In accordance with UK Health Research Authority guidance, formal research ethics committee approval and individual patient consent were not required. The project was formally approved and registered with both the Clinical Audit & Effectiveness Team and the Quality Governance Team at the Royal Free London NHS Foundation Trust. The evaluation was approved locally through the trust’s clinical governance processes and conducted in line with the principles of the Declaration of Helsinki.

### Data collection

Electronic medical records were reviewed to extract demographic information, tumour histology, scan indication, findings, and subsequent clinical management. Each scan was classified as either MDT endorsed or individually requested based on documentation in MDT meeting minutes and referral records.

### Scan indication

The clinical indication for each I-123 scan was determined retrospectively from referral documentation and contemporaneous clinical records. Recorded scan indications were grouped into predefined clinically meaningful categories, including biochemical concern, imaging-based concern, suspected or known metastatic disease, and risk stratification in the context of clinical uncertainty.

Where more than one clinical factor was present, a single primary indication was assigned based on the dominant reason documented for requesting the scan.

### Scan acquisition and preparation

I-123 scintigraphy was performed according to departmental protocols as described in nuclear medicine reports. Imaging protocols typically included whole-body planar imaging, with SPECT or SPECT/CT performed. Scans were performed at standard time intervals following tracer administration in line with contemporaneous departmental practice. Patients were prepared using either thyroid hormone withdrawal or recombinant human TSH stimulation according to contemporaneous clinical practice; preparation method was not consistently documented for all scans.

### Outcome definitions

Management outcomes were analysed hierarchically. Initial outcomes captured diagnostic actions triggered by I-123 findings, including requests for further imaging. A secondary analysis assessed definitive therapeutic management changes after completion of downstream investigations. A definitive management change was defined as additional radioiodine therapy, surgery or local excision, or modification of thyroid hormone suppression strategy. An absence of a definitive management change was recorded where no alteration to treatment occurred.

### Statistical analysis

Data were analysed using descriptive statistics. Proportions were expressed as percentages with 95% confidence intervals (CIs) calculated using the Wilson score method for binomial data. Differences in management change between MDT and non-MDT scans were compared using Fisher’s exact test. Statistical significance was defined as *P* < 0.05. A secondary descriptive analysis incorporated outcomes after completion of downstream investigations to assess overall management impact.

## Results

### Study cohort

Fifty-five I-123 scans from 51 patients were analysed. The cohort was predominantly female (68.6%) with a mean age of 44.4 years (range 18.9–82.7). Histology comprised papillary carcinoma in 44 patients (86.3%), follicular in 5 (9.8%), and Hürthle cell in 2 (3.9%) (Table 1).

**Table 1:** Patient characteristics.

Characteristic	*n*	%
Female	35	68.6
Male	16	31.4
Papillary carcinoma	44	86.3
Follicular carcinoma	5	9.8
Hürthle cell carcinoma	2	3.9

### Scan indications

I-123 scintigraphy was performed for a range of clinical indications. The most common indications were biochemical concern (elevated thyroglobulin or thyroglobulin antibodies), concern arising from prior radioiodine post-treatment imaging, and evaluation of suspected or known recurrent or metastatic disease. Less common indications included concerning ultrasound findings and new symptoms. In a small number of cases, the indication was unclear from the documentation. Indications are summarised in Table 2.

**Table 2:** Primary indications for I-123 scintigraphy.

Indication	*n*	%
Biochemical concern (raised Tg/TgAb)	25	45.5
Concern on I-131 exit scan	10	18.2
Structural imaging concern (ultrasound or lymph node findings)	6	10.9
Suspected or known metastatic or recurrent disease	7	12.7
Previous extensive disease	2	3.6
New symptoms	1	1.8
Unclear	4	7.3

### Scan findings and initial diagnostic actions following I-123 scintigraphy

Across all scans, 21 of 55 (38.2%) demonstrated new or abnormal findings, while 34 scans (61.8%) showed no abnormality.

Following I-123 imaging, initial diagnostic actions were undertaken in 23 of 55 scans (41.8%), while no immediate action was taken in 30 scans (54.5%). In 2 scans (3.6%), documentation of subsequent action was unclear.

When diagnostic action followed I-123 scintigraphy, the most frequent actions were further imaging (12 cases; 21.8%), additional radioiodine therapy (5; 9.1%), surgery or local excision (4; 7.3%), and relaxation of TSH suppression targets (2; 3.6%). These initial diagnostic actions are summarised in Table 3.

**Table 3:** Initial diagnostic actions following I-123 scintigraphy.

Initial diagnostic action	*n*	% (of 55)	95% CI (Wilson)
No immediate action	30	54.5	41.5–67.0
Further imaging	12	21.8	13.0–34.4
Additional radioiodine therapy	5	9.1	3.9–19.6
Surgery/local excision	4	7.3	2.9–17.3
Relaxed TSH suppression target	2	3.6	1.0–12.3
Unclear	2	3.6	1.0–12.3

### Influence of multidisciplinary team (MDT) discussion on initial diagnostic actions

Twenty scans (36.4%) were requested following MDT discussion, whereas 29 scans (52.7%) were requested by individual clinicians. In six cases (10.9%), documentation of the requesting source was unclear.

Initial diagnostic actions following I-123 imaging occurred in 10 of 20 MDT-requested scans (50.0%) compared with 9 of 29 scans (31.0%) requested by individual clinicians. Although this difference did not reach statistical significance (*P* = 0.37, Fisher’s exact test), MDT-endorsed scans more frequently prompted further diagnostic investigation. Among scans with unclear requester documentation, initial diagnostic action occurred in 4 of 6 cases (66.7%). Of note, in two scans requested by individual clinicians, it was unclear if there was any subsequent change in management. Initial diagnostic actions by requester category are summarised in Table 4.

**Table 4:** Initial diagnostic actions following I-123 scintigraphy by requester.

Request type	Total scans	Management change: yes		Management change: no		*P* (Fisher’s)
		*n* (%)	95% CI (Wilson)	*n* (%)	95% CI (Wilson)	
MDT	20	10 (50.0%)	29.9–70.1	10 (50%)	29.9–70.1	
Individual clinician	29*	9 (31.0%)	17.3–49.2	18 (62.1%)	44.0–77.3	0.37 (ns)
Unclear requester	6	4 (66.7%)	30.0–90.3	2 (33.3%)	9.7–70.0	
Total	55	23 (41.8%)	29.7–55.0	30 (60%)	41.5–67.0	

* In two scans requested by individual clinicians, management outcomes were indeterminate.

### Definitive management outcomes after completion of downstream investigations

**Figure 1:**
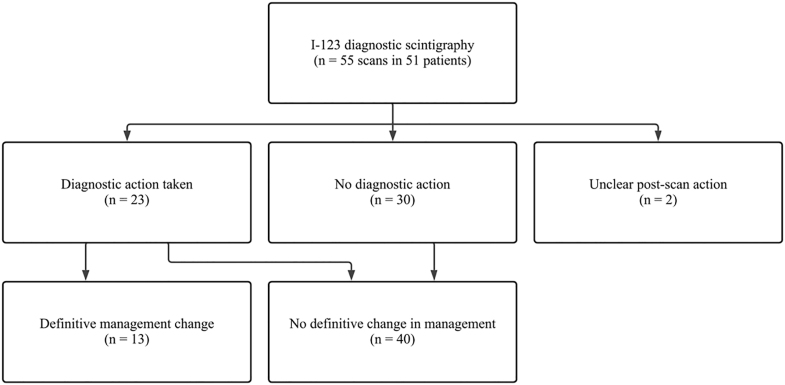
Hierarchy of outcomes following iodine-123 (I-123) diagnostic scintigraphy in patients undergoing follow-up for differentiated thyroid cancer. Initial diagnostic actions and definitive management outcomes are shown. In two scans, post-scan management documentation was unclear and these cases are shown as a separate terminal category.

A flow diagram illustrating the hierarchy of outcomes following I-123 scintigraphy, from initial diagnostic action to definitive management outcome, is shown in Fig. 1.

When management outcomes were reassessed after completion of downstream investigations prompted by I-123 imaging, no definitive change in management occurred in 40 of 55 scans (72.7%). A definitive management change occurred in 13 of 55 scans (23.6%). In 2 scans (3.6%), the final management impact remained unclear due to incomplete documentation.

Definitive management changes included additional radioiodine therapy (6 scans; 10.9%), surgery or local excision (5; 9.1%), and relaxation of TSH suppression targets (2; 3.6%). These outcomes are summarised in Table 5.

**Table 5:** Definitive management outcomes after completion of downstream investigations.

Outcome	*n*	% (of 55)	95% CI (Wilson)
No change in management	40	72.7	59.8–82.7
Additional RAI therapy	6	10.9	5.1–21.8
Surgery/local excision	5	9.1	3.9–19.6
Relaxed TSH suppression target	2	3.6	1.0–12.3
Unclear	2	3.6	1.0–12.3

### Definitive management outcomes by requester

After incorporation of downstream investigation outcomes, definitive management change occurred in 20.0% (4/20) of MDT-requested scans, 20.7% (6/29) of individually requested scans, and 50% (3/6) of scans with unclear request source. This showed remarkably similar outcomes for both the MDT and clinician requested scans, with only ∼ one in five scans leading to any change in management. These results are summarised in the Table 6.

**Table 6:** Definitive management outcomes by requester (after incorporation of downstream investigations).

Request type	Management change: yes		Management change: no		Management change: unclear		*P* (Fisher’s)
	*n* (%)	95% CI (Wilson)	*n* (%)	95% CI (Wilson)	*n* (%)	95% CI (Wilson)	
MDT	4 (20.0%)	8.1–41.6	16 (80%)	58.4–91.9	0 (0%)	0–16.1	
Individual clinician	6 (20.7%)	9.8–38.4	21 (72.4%)	54.3–85.3	2 (6.9%)	1.9–22.0	1 (ns)
Unclear requester	3 (50%)	18.8–81.2	3 (50%)	18.8–81.2	0 (0%)	0–39.0	
Total	13 (23.6%)	14.4–36.4	40 (72.7%)	59.8–82.7	2 (3.6%)	1.0–12.3	

## Discussion

This single-centre service evaluation examined the clinical utility of I-123 diagnostic scintigraphy in the follow-up of patients with differentiated thyroid cancer, with particular emphasis on its impact on downstream management. Although I-123 scans identified abnormal findings and frequently prompted further investigation, fewer than one quarter of scans ultimately resulted in a definitive change in management once subsequent imaging and clinical review were completed. This reduction highlights that many early scan-driven actions reflected interim investigations rather than true alterations in therapeutic direction.

A key contribution of this study is the explicit distinction between diagnostic actions and durable therapeutic change. While new or abnormal findings were identified in 38.2% of scans and 21.8% prompted further imaging, the majority of patients did not experience any alteration in definitive management. It is important to acknowledge that a diagnostic investigation may have clinical value even when it does not lead to a change in management, for example by providing reassurance or excluding clinically significant disease; however, this potential benefit must be weighed against the associated radiation exposure, patient inconvenience, and cost of I-123 scintigraphy. This distinction is clinically important, as previous studies evaluating diagnostic radioiodine imaging have often reported surrogate outcomes, such as scan positivity or subsequent investigation, and have not always distinguished these from definitive therapeutic change.

Importantly, I-123 scans were requested for heterogeneous clinical indications, reflecting real-world follow-up practice rather than a single predefined scenario. Explicitly describing the reasons for scan request provided transparency regarding referral patterns and allows readers to judge the applicability of these findings to their own clinical settings, without implying indication-specific differences in diagnostic yield.

The comparison between MDT-endorsed scans and those requested by individual clinicians provides further insight into how I-123 imaging is used in practice. Although initial diagnostic actions occurred numerically more often following MDT-endorsed scans, this difference was not statistically significant and did not translate into a higher rate of definitive management change after downstream investigations were completed. This suggests that MDT discussion may influence diagnostic caution and investigation pathways without necessarily altering ultimate treatment decisions.

Due to the modest diagnostic yield, costs, inconvenience, and radiation exposure, I-123 scans are far from a perfect test in following up post-treatment differentiated thyroid cancer patients. When outcomes after subsequent imaging were considered, fewer than one in four scans ultimately led to a definitive change in management. This finding aligns with current national and international guidance, including NICE and American Thyroid Association recommendations, which advocate selective rather than routine use of diagnostic radioiodine imaging in the follow-up of differentiated thyroid cancer.

This study has limitations, including its retrospective design, single-centre setting, and modest sample size. Although a small number of patients underwent more than one scan, analyses were performed at the scan level. A sensitivity analysis restricted to the first scan per patient was considered but not performed due to small numbers and the descriptive nature of this study. Incomplete documentation of some clinical variables over a long study period limited more granular subgroup analysis. However, these limitations reflect real-world clinical practice and do not detract from the central observation regarding the downstream impact of I-123 imaging on management decisions.

Taken together, these findings underscore the importance of careful case selection when using I-123 in follow-up of patients post-thyroidectomy. Given the associated radiation exposure, patient inconvenience, and cost, I-123 scintigraphy should be targeted to clinical scenarios where it is most likely to provide actionable information rather than used routinely.

## Conclusion

I-123 scintigraphy can influence clinical decision-making in the follow-up of patients with differentiated thyroid cancer, most commonly by prompting further diagnostic investigation. However, when downstream outcomes are considered, definitive changes in management occur in a minority of cases, supporting a selective rather than routine role for I-123 imaging in follow-up.

## Declaration of interest

The authors declare that there is no conflict of interest that could be perceived as prejudicing the impartiality of the research reported.

## Funding

This research did not receive any specific grant from any funding agency in the public, commercial, or not-for-profit sector.
